# Stuck in the mud: time for change in the implementation of cognitive training research in ageing?

**DOI:** 10.3389/fnagi.2014.00043

**Published:** 2014-03-17

**Authors:** Courtney C. Walton, Loren Mowszowski, Simon J. G. Lewis, Sharon L. Naismith

**Affiliations:** Healthy Brain Ageing Program, Brain and Mind Research Institute, University of SydneySydney, NSW, Australia

**Keywords:** cognitive training, cognitive remediation, healthy brain ageing, dementia, research methodology

Over the past two decades, within the field of healthy ageing and dementia prevention there has been a substantial growth of interest in the potential of cognitive training (CT) interventions (see Figure 1). Whilst various studies have employed different methodologies, generally the term refers to programs which provide theoretically driven skills and strategies, involving guided practice on tasks reflecting specific cognitive functions (Mowszowski et al., 2010). The focus of such interventions is to improve functioning of particular cognitive skills such as memory, working memory, attention, and executive functions, as decline in these or other cognitive domains may lead to functional impairment in day-to-day activities as well as contribute to reduced quality of life and disability (Salthouse, 2004). Improvements in these cognitive abilities may lead to more effective or independent functioning and may be instigated through various CT approaches including repetitive computerized exercise, pen and paper tasks, and clinically-driven strategy learning.

**Figure 1:**
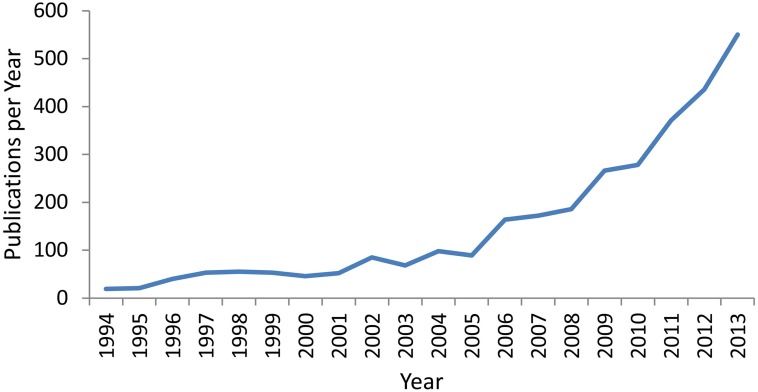
**Number of publications per year over the last 2 decades containing the terms “Cognitive Training,” “Cognitive Remediation,” or “Cognitive Rehabilitation” in the title, abstract, or key-words**. Data was extracted from the Scopus Database on 14/02/2014.

The importance of further exploring this method for preventing or delaying decline is clear, with the growth of the world's older adult population in the first half of this century anticipated to be staggering. With this comes a significant increase in the incidence of dementia and age-related cognitive decline. Indeed, it has been predicted that by 2050 in the United States alone, there will be 13.8 million people suffering from Alzheimer's disease (Hebert et al., 2013). In response, severe consequences for health service costs in addition to lowered quality of life in sufferers and their family members or carers will become key issues for our society (Comas-Herrera et al., 2007).

Ultimately, it has been proposed that CT should be used as a preventative technique for delaying, reducing or preventing cognitive decline, or as a method of restoring function and coping with deficits (Naismith et al., 2009; Mowszowski et al., 2010). CT has numerous strengths due to its relatively easy implementation, as compared to pharmacological means, and high potential for at-home personalized use or clinical facilitation. As such, in addition to other methods, investigating this potential means of dementia prevention and delayed decline should be of utmost importance in current thinking and should represent an international health-related research priority. However, despite the clear need for thorough, effective research, CT trials to date have been relatively mixed in regards to fulfilling the rigorous scientific standards required to warrant implementation and investment in such health interventions.

Despite this, the research has undoubtedly grown in a positive direction from early beginnings. Numerous well conducted trials have been able to show benefits in CT that can translate to non-practiced neuropsychological tasks, that are still evident at follow-up testing, and carry over onto non-cognitive functional measures (for reviews see: Zelinski, 2009; Gates et al., 2011; Kueider et al., 2012; Jak et al., 2013). Anecdotally, many clinicians observe that participants, carers, and family-members often report a subjective sense of improvement to functioning in addition to objective improvements on formal outcome measures. Such subjective improvements have been empirically demonstrated, for example in knowledge and use of memory strategies (Kinsella et al., 2009; Naismith et al., 2013), perceptions of cognitive abilities (Smith et al., 2009) and well-being (Belleville et al., 2006).

Furthermore, our understanding of the theory underlying the effectiveness of CT is growing. Evidence for the role of neuroplasticity resulting from multiple types of training and how it can be used is now more clearly apparent (Cramer et al., 2011; Park and Bischof, 2013; Patel et al., 2013). Additionally, our understanding of the relevance and applicability of cognitively demanding experience as a contributor to protection against dementia (i.e., cognitive reserve) is expanding (Stern, 2002; Valenzuela et al., 2007; Valenzuela and Sachdev, 2009). However, there still remains no gold standard of evidence to confirm how best CT can be effective. It is clear that many researchers believe that the lack of a *consistently* strong finding across studies may likely reflect methodological differences, given the relatively robust underlying neurobiological theory and the many positive research findings that have been found.

A number of recent reviews which have extensively analyzed the literature clearly show that the heterogeneity in methodology and content of CT studies in addition to low quality trials is leading to exceedingly difficult synthesis of data and interpretation of findings. For example, recent work which analyzed the evidence for CT in Alzheimer's disease was unable to make reliable conclusions of the literature based primarily on the low quality of current studies (Bahar-Fuchs et al., 2013). Such a finding may relate specifically to the particular patient group under investigation, yet other reviews of differing populations such as healthy older adults and those with mild cognitive impairment which have indeed suggested more positive results, still conclude that there is much work to be done in terms of methodological heterogeneity across studies (e.g., Mowszowski et al., 2010; Gates et al., 2011; Kueider et al., 2012; Rabipour and Raz, 2012; Huckans et al., 2013; Jak et al., 2013; Reijnders et al., 2013).

Various issues regarding CT research can be provided as possible reasons for the common lack of consensus across studies. Briefly, in terms of experimental design, such issues arise from a lack of double-blinded, randomized active-controlled trials. Such methodology should ideally be required for publication of findings, in accordance with appropriate reporting standards (Moher et al., 2001). Trials should contain sham training whereby clinician interaction and participant expectation effects can be reliably matched. While a push toward the use of active controls in trials is apparent, often the active element is not sufficient, with patients engaging in activities possibly too simple to match for clinical interaction and expectancy effects (Boot et al., 2013). For example, sham training may involve low-level non-adaptive training in very simple CT tasks that would not be expected to elicit any meaningful change, yet more accurately represent a behavioral placebo (Brehmer et al., 2012). In addition, studies often lack sufficiently powered samples, complete training over too short a time period, and do not allow for a significant follow up period of testing.

Communication of such methodology must also be improved, as inappropriate or unclear terminology reported for remediation techniques is common, in addition to inadequate details of training procedures. Indeed, this problem may be evident in much of the non-pharmacological literature (Hoffmann et al., 2013). For example, cognitive outcome measures should entail standardized neuropsychological tasks, which can be interpreted by trained clinicians and be compared to a plethora of literature for review and meta-analytic techniques. Additionally, more informative and sensitive measures of functional change need to be developed and implemented. The construction of further standardized questionnaires relating to self-assessed functional and cognitive changes may be valuable for cross-study comparison and is thus worthy of increased research interest.

Although a number of these criticisms are regularly reported as limitations in many research articles and reviews, insufficient changes have been made to address these problems. In this way, it would appear that we are moving further from discovering what is most effective by diluting the literature with often incomparable studies. Thus, we are blocking our progression toward consistently solid evidence for CT efficacy and uncovering what is hoped are tangible and very real benefits for the ageing population. In addition to the abovementioned limitations, we suggest the following areas of research may enhance future study quality and translational ability.

Further areas of interest in improving the validity of findings include understanding the time-course and sustainability of CT-related improvements, and work is currently being conducted to explore this in more detail (Lampit et al., 2013). Many studies have been able to show sustained results at follow-up testing (Mahncke et al., 2006; Li et al., 2008; Brehmer et al., 2012; Rebok et al., 2014), but the processes underlying the duration of effects are not well understood. Another area of importance yet to be fully explored is the role of individual differences in training gains. Factors including cognitive reserve and ability (e.g., education, IQ, employment), relevant personality traits (e.g., locus of control, self-efficacy), pre-training cognitive performance, diet, smoking and alcohol intake, or sub-threshold depressive and anxious symptoms are likely to play an important role in how different participants respond to training. Early exploration of this area appears to support the notion that such factors should be included as possible mediating variables, as they may impact on individual levels of training efficacy. Moreover, recent work has suggested that such individual differences may be helpful in predicting which patients may benefit most from CT, thus enabling a more targeted approach (Jaeggi et al., 2013; Rebok et al., 2013; Willis and Caskie, 2013).

A key issue in ageing research in particular is developing stronger longitudinal evidence for protective benefits in “at-risk” groups such as patients with mild cognitive impairment and late-life depression (Gates et al., 2011; Naismith et al., 2011). For example, future studies should aim to clearly investigate any differences in the trajectory of cognitive decline between “at-risk” groups who undergo CT programs, compared to those who do not receive such interventions.

Further interest involves understanding the most appropriate delivery of CT interventions. While many researchers and clinicians favor a strictly computerized “drill-like” program, some studies suggest that the implementation of concurrent psycho-education or similar programs may enhance CT-related gains, either by improving understanding (i.e., education: cognition, modifiable risk factors for healthy brain ageing) or by boosting program engagement (Norrie et al., 2011). Crucially, such a format may improve the acceptance and tolerance of intensive training in participants leading to better outcomes. Concomitant strategy training may also booster the observed effects of standard computerized CT (Sohlberg and Mateer, 2001; Wilson, 2008; Naismith et al., 2011, 2013). Such methodology is especially relevant in heterogeneous clinical populations as compared with healthy populations, and has important implications for effective clinical practice.

Ultimately, the goal of CT research should be to implement such programs within the community or clinical setting. Understanding how best to make such programs accessible and feasible in clinical, disadvantaged or hard-to-reach populations is complex, and may require involvement of local healthcare or public health services to facilitate translation. One area of growth may be the use of e-health platforms and implementation in aged-care facilities. To this note, the literature lacks substantial detailed evidence for the cost-effectiveness of these programs, and such research would be advantageous.

## Conclusion

In order to progress the field of CT to the methodological caliber of biomedical and pharmacological interventions, systematic, structured guidelines for the implementation of CT programs are warranted. We suggest that the field would benefit from an international meeting of CT researchers and clinicians, to discuss how best to resolve some of these issues, as has been implemented in other fields of research (e.g., Green et al., 2004). Primary importance should be directed to work-shopping collaboratively, with the common goal of formulating a set of systematic concrete guidelines, to facilitate consistency across studies leading to better synthesis of findings. This would inform future research as to how most effectively assess the efficacy of CT, and lead to more rapid transfer of research knowledge into clinical and community settings. Such a meeting could also involve training forums, so that clinicians are appropriately trained and are utilizing the available evidence. Whilst it may take some time to establish and implement such guidelines, it appears the time has arrived for more active engagement in addressing what have been described as limitations in the literature for too long. It is our hope that the scientific community involved in CT research can work together to confront this issue, so that this important and worthwhile field can grow upwards rather than outwards.

## Author contributions

Courtney C. Walton prepared the original manuscript. Loren Mowszowski, Simon J. G. Lewis, and Sharon L. Naismith edited and gave final approval for publication. All authors are accountable for this work.
